# Economic analysis of three interventions of different intensity in improving school implementation of a government healthy canteen policy in Australia: costs, incremental and relative cost effectiveness

**DOI:** 10.1186/s12889-018-5315-y

**Published:** 2018-03-20

**Authors:** Kathryn L. Reilly, Penny Reeves, Simon Deeming, Sze Lin Yoong, Luke Wolfenden, Nicole Nathan, John Wiggers

**Affiliations:** 1 School of Medicine and Public Health, University of Newcastle, Callaghan, NSW 2308 Australia; 2 Hunter Medical Research Institute, Newcastle, NSW 2300 Australia; 3 Priority Research Centre for Health Behaviour, University of Newcastle, Callaghan, NSW 2308 Australia; 4 Hunter New England Population Health, Locked Bag 10, Wallsend, NSW 2287 Australia

**Keywords:** Economic evaluation, Implementation strategies, Healthy eating policies, Childhood obesity

## Abstract

**Background:**

No evaluations of the cost or cost effectiveness of interventions to increase school implementation of food availability policies have been reported. Government and non-government agency decisions regarding the extent of investment required to enhance school implementation of such policies are unsupported by such evidence. This study sought to i) Determine cost and cost-effectiveness of three interventions in improving school implementation of an Australian government healthy canteen policy and; ii) Determine the relative cost-effectiveness of the interventions in improving school implementation of such a policy.

**Methods:**

An analysis of the cost and cost-effectiveness of three implementation interventions of varying support intensity, relative to usual implementation support conducted during 2013–2015 was undertaken. Secondly, an indirect comparison of the trials was undertaken to determine the most cost-effective of the three strategies. The economic analysis was based on the cost of delivering the interventions by health service delivery staff to increase the proportion of schools ‘adherent’ with the policy.

**Results:**

The total costs per school were $166,971, $70,926 and $75,682 for the high, medium and low intensity interventions respectively. Compared to usual support, the cost effectiveness ratios for each of the three interventions were: A$2982 (high intensity), A$2627 (medium intensity) and A$4730 (low intensity) per percent increase in proportion of schools reporting ‘adherence’). Indirect comparison between the ‘high’ and ‘medium intensity’ interventions showed no statistically significant difference in cost-effectiveness.

**Conclusions:**

The results indicate that while the cost profiles of the interventions varied substantially, the cost-effectiveness did not. This result is valuable to policy makers seeking cost-effective solutions that can be delivered within budget.

## Background

The prevalence of overweight and obesity in children of high income countries has become a major health concern. Globally in 2013, approximately 24% of children were classified as overweight or obese, an increase of almost 17% since 1980 [1]. Similarly, Australian data indicates that the prevalence of overweight and obesity in children has doubled over recent decades [2, 3]. Childhood obesity contributes to a significant financial burden on the healthcare system, with over 50% of obese children continuing to be so as they move into adulthood [4]. A recent systematic review estimated that obesity accounted for between 0.7% and 2.8% of a country’s total healthcare expenditure [5]. As such, interventions to prevent excessive weight gains have been identified as a priority by governments globally.

Recent reviews and trials suggest that improving the relative availability of healthy foods, particularly in schools, is effective in reducing the prevalence of child overweight and obesity [6] and/or its behavioral determinants [7]. For example a recent review by Mayne et al.. (2015) found that school food environments that restrict sugary foods and beverages or higher fat foods, and/or had increases in availability of milk and fruits/vegetables reported favorable impacts on purchases or self-reported food consumption [7]. Likewise, in a trial to increase the availability of healthy food products and restrict the availability of unhealthy products reported by Wolfenden et al.. (2017), student purchases from intervention school canteens were significantly lower in total fat (− 132.32 kJ; 95% CI − 280.99 to 16.34; *p* = 0.080) with trends towards improvement in sodium (− 46.81 mg; 95% CI − 96.97 to 3.35; *p* = 0.067) and energy intake (− 132.32 kJ; 95% CI − 280.99 to 16.34; p = 0.080) [8]. A review by Katz et al (2008) also found that interventions that include improvements to the school nutrition environment are effective in achieving weight reduction in the school setting [6].

Evidence from systematic reviews also suggests that obesity prevention interventions delivered in schools are cost-effective [9, 10]. A recent review (2014) of the cost-effectiveness of childhood obesity prevention programs identified three school based programs that were cost-effective [9]. Of these studies two included, amongst other strategies, changes to the availability of food, suggesting that the inclusion of food availability policies may contribute to cost-effective obesity prevention [11, 12].

Many high income countries have introduced nutrition policies in schools that support the provision of healthier food and beverage options and restrict unhealthy options in line with national dietary guidelines [13–15]. Despite the introduction of such policies, the extent of school adherence to such policies is limited. For example, results of the 2012 School Health Policies and Practices Study (SHPPS) in the United States found that almost 60% of secondary schools did not adhere to recommended nutrition standards by selling energy dense nutrient poor foods, such as chocolate, pastries, salty snacks and sweetened drinks [16]. Similarly, a recent review (2016) of the adoption of healthy school food policies in Australian schools found that adherence with such policies in canteens was low [17]. Without widespread school implementation of such policies, their intended benefits at the population level are unlikely to be achieved. Such findings suggest a need for research regarding strategies to increase school adherence to school food availability policies and recommendations.

Three such implementation studies have investigated the effectiveness of strategies to increase schools’ implementation of nutrition initiatives broadly, and of policies and practices regarding the availability of food in school canteens and food service settings specifically [8, 18, 19]. The trials were conducted in a single region of Australia, in the same time period (2013–15), involved common outcome measures (food availability/ policy adherence) and assessed interventions involving differing modalities and intensity. Two of the trials were found to be effective [8, 18] with the third approaching statistical significance (*p* = 0.06) [19]. No economic analyses of the trials were reported.

To the author’s knowledge, no evaluations of the cost or cost-effectiveness of other interventions to increase school implementation of food availability policies have been reported. In the absence of such information, government and non-government agency decisions regarding the nature and extent of investment required to enhance school implementation of such policies is unsupported by relevant evidence.

To address the evidence gap regarding the cost and cost effectiveness of interventions to increase school adherence with food availability policies, an economic evaluation was conducted of the three recently reported intervention trials [8, 18, 19]. Specifically, the study sought to; i) Determine the cost and cost-effectiveness of each of the three interventions in improving school implementation of a government healthy canteen policy and; ii) Determine the relative cost-effectiveness of the three interventions in improving school implementation of such a policy.

## Methods

### Study design

Two separate but related analyses were undertaken. First, a within-trial evaluation of the cost and cost-effectiveness of three implementation interventions, relative to usual implementation support, was undertaken. Usual implementation support involved government-provided training for schools to develop action plans targeting a variety of healthy eating practices, including healthy food availability in school canteens [20]. Second, a between-trial comparison was undertaken to determine the most cost-effective of the three intervention strategies in increasing school implementation of the policy.

The studies adopted a health service delivery perspective and involved analysis of the direct costs to health services of providing implementation support. Health services in the state of New South Wales (NSW) Australia are a provider of support for school implementation of the healthy school canteen policy. Health services, alongside school-aged children and families, are also a significant potential beneficiary of the interventions in terms of the benefits that may accrue from improved nutrition, such as net savings in healthcare utilisation. The base year for all analyses was 2015 with costs reported in Australian dollars.

### Context

In Australia, children are able to purchase foods and drinks during recess and lunch time over the counter from a canteen physically located on school premises. All Australian states and territories have introduced healthy canteen policies that utilize a ‘traffic light’ system to promote healthy foods and restrict the sale of less healthy foods [21]. In NSW specifically, the government mandated a healthy school canteen policy for all government primary and secondary schools in 2005 [15]. The policy categorized canteen menu items based on their nutritional value [15]. To adhere with the policy, school canteens were required to fill at least 50% of the menu with ‘green’ (healthier) foods, limit the availability of ‘amber’ (less healthy) foods and restrict the sale of ‘red’ (poor nutritional value) foods. In 2007, a ‘Sugar Sweetened Drink Ban’ was introduced which bans the sales of sugar sweetened drinks based on their nutrient content [15]. School support officers employed by local health services across the state provided policy implementation support to schools.

### Trial design and setting

Three randomized controlled trials were conducted involving primary schools in one region of NSW, Australia [8, 18, 19]. The region covers a large geographic area (more than 130,000 km2) and consists of a socioeconomically and demographically diverse population of approximately 112,000 children aged 5–12 years [22].

### Participants and recruitment

Primary schools (with students 5 to 12 year olds) were eligible to participate in the three trials if they had a canteen open at least one day per week. Schools enrolling both primary and secondary students and schools catering exclusively for children requiring specialist care were excluded from the trials. Additional eligibility criteria for the ‘high intensity’ and ‘low intensity’ trials included only government schools with menus not adherent to the healthy canteen policy. For all three trials, school principals were contacted via phone or email and invited to participate in the study.

### Implementation interventions and outcomes

All three randomized controlled trials aimed to enhance school implementation of the government healthy canteen policy by addressing known barriers to the implementation of the policy [21, 23, 24]. The three trials employed intervention strategies of varying intensity defined according to three levels of labor support provided by school support officers and number of strategies included (‘high’, ‘medium’ or ‘low’). Intervention strategies for the ‘high intensity’ and ‘medium intensity’ intervention were guided by the Theoretical Domains Framework [8, 18] whilst the ‘low intensity’ intervention was designed using Control Theory [19]. (Table 1).

**Table 1:** Summary of strategies and costs for the three trials

Strategies	Description and/or cost components	High intensity intervention [8] Trial registration: ACTRN12613000311752	Medium intensity intervention [18] Trial registration: ACTRN12614001148662	Low intensity intervention [19] Trial registration: ACTRN12613000543785
Percentage of schools for each trial that provided menus for audit at follow-up.		81%	96%	74%
1. Policy implementation support	The support officer provided targeted advice to overcome common barriers to policy implementation and to encourage canteen managers to review progress against action plans.	$151,062	$65,111	$71,128
2. Executive support	School principals were asked to communicate support for policy implementation and maintenance to teachers, parents, students and canteen managers during staff meetings, in newsletters, and assemblies.	Cost included in support staff wages in Policy Implementation		
3. Consensus processes	Meetings between support staff and canteen staff were held to discuss and reach consensus regarding the policy, how best to implement it and to develop local canteen action plans to co-ordinate implementation tasks.	Cost included in support staff wages in Policy Implementation		
4. Training	Canteen managers, canteen staff and parent representatives were invited to attend a training workshop (five hours) with the aim of providing education and skill development in the policy, nutrition and food label reading, canteen stock and financial management, pricing and promotion, and change management. Training combined didactic and interactive components including opportunities for self-assessment, role play and facilitator provided feedback. Training was facilitated by support staff.	$6376	$833	N/A
5. Tools and resources	Provision of “Canteen Resource Kit” containing various printed and electronic instructional materials, including electronic menu and pricing templates, and a poster-sized checklist that prompted canteen managers to regularly review their canteen practices. Canteen managers also received kitchen equipment to the value of AUD$100.	$4781	$2959	N/A
6. Academic detailing	School canteen visits were conducted one and three months post canteen manager training to enable support officers to observe the operational canteen environment, provide feedback, and assist with problem solving barriers to policy implementation.	Cost included in support staff wages in Policy Implementation	N/A	N/A
7. Recognition	Schools with a menu assessed as adhering to the policy (i.e. greater than 50% ‘green’ items and no ‘red’ or ‘banned’ items) were acknowledged.	$27	$0	N/A
8. Performance monitoring and feedback	Menu reviews were conducted (unless menus were unchanged) and the results were used to compile written feedback reports to the canteen manager and school principal. Costs; collection of menus, conduct audits and generate feedback reports	$4428 (4/school)	$2024 (2/school)	$4554 (4/school)
9. Marketing strategies	Quarterly project newsletters communicated key messages, provided information and case studies of successful implementation approaches to common barriers.	$298	N/A	N/A
Total cost		$166,971	$70,926	$75,682
Total cost / school		$4771	$2216	$2102

### High intensity support trial

The trial involved 35 intervention and 35 control schools over a 12–14 month period. The intervention consisted of a multi-strategic approach involving policy implementation support in conjunction with executive support, consensus processes, staff training, provision of tools and resources, academic detailing, recognition, performance monitoring and feedback and marketing strategies. The intervention also involved intensive on-going support provided by local health district project officers which involved bi-monthly school visits with the canteen manager, principal meetings and school parent representative group (P&C meetings) presentations.

### Medium intensity support trial

The trial involved 28 intervention and 25 control schools over a 9 month period. Implementation strategies used in the ‘high intensity’ support trial were included such as executive support, the provision of tools and resources, staff training, performance monitoring and feedback, and recognition in conjunction with a less expensive mode of on-going support via text messaging as oppose to school onsite-visits. Canteen managers received two support contacts per school term via text messages which provided targeted advice to overcome common barriers to policy implementation and encouraged canteen managers to review progress against their action plan.

### Low intensity support trial

The trial involved 36 intervention and 36 control schools over a 12 month period. Implementation support designed to test the effectiveness of a low intensity, lower cost strategy, including canteen menu audits to assess compliance with the State policy and subsequent provision of feedback regarding the content of canteen menus via a written report and telephone call each school term (four times) was delivered.

### Trial outcome data collection procedures and measures

For the three trials, outcome data were collected at baseline and immediately following completion of each of the interventions. Full details of menu audit procedures are reported elsewhere [8, 18, 19, 25]. In brief, schools provided copies of their current canteen menu for audit by a dietitian, trained in menu assessment, blinded to group allocation. Using a menu assessment protocol, dietitians classified all food and beverage menu items as either ‘green’, ‘amber’, ‘red’ or ‘banned’ according to the policy criteria and determined menu composition by calculating the percentage of the total number of items on the menu that were ‘green’, ‘amber’, ‘red’ or ‘banned’. The primary trial outcomes of all three trials was the proportion of canteen menus that (i) did not contain foods or beverages restricted for sale (‘red’/ ‘banned’), and, (ii) where healthy canteen items (‘green’) represented more than 50% of listed menu items [8, 18, 19]. For the purposes of the economic analysis, and in order to have a single comparable effect measure, we combined these two trial outcomes and calculated a measure of full compliance of the policy for all interventions.

### Cost data collection procedures and measures

A retrospective economic analysis was undertaken based on the cost of delivering the interventions by health service delivery staff. For each of the three trials, project management records relating to intervention delivery included recording of costs regarding (where relevant): i) school support staff salary costs for support contacts with school principals and canteen staff; menu collection, assessment and generation of feedback reports; canteen staff training and workshop co-ordination; and for project management; ii) canteen staff training expenses such as venue hire, catering and reimbursement of canteen staff expenses to attend workshops; iii) the provision of canteen equipment and the printing of resources assisting in the financial management and development of menus for canteen staff and; iv) health service overheads such as administration support, telephone and car usage.

In terms of school support staff salary costs, due to the number and diversity of seniority of personnel involved (six staff across the three trials), school support staff time was costed at the mid-point in the relevant pay scale, whereas project manager time was actual manager salary (two managers across the three trials). Salary costs for conducting menu audits and coordination of canteen staff training workshops was based on the relevant casual salary rate of employed staff. Venue hire costs for canteen staff training workshops were the actual rates charged, or if held on health service premises at no cost, the external rate for hire was included. Consumable costs such as catering, printing, stationary and canteen equipment were measured directly and valued using market prices.

For control schools, it was assumed that no additional costs were incurred in implementing their usual canteen management practices.

## Analytical methods

All analyses were undertaken using Microsoft Excel software 2013. Research related costs together with intervention development and set up costs were excluded from the analysis to achieve a focus on the costs and cost-effectiveness of delivering the interventions only. As the analysis was taken from a health service delivery perspective, costs to canteen managers, principals or schools, including opportunity costs were not assessed.

### Within-trial cost and cost effectiveness

Incremental costs and costs per school were calculated for all three interventions. The average cost per school for each intervention was determined by summing the intervention delivery costs and dividing the total cost by the number of intervention schools. Incremental cost-effectiveness ratios (ICERs) were calculated within trials and expressed as costs per percentage point increase in the proportion of schools adherent with the policy. Uncertainty intervals around each of the ICERs were derived from the confidence intervals around the ‘adherence’ outcome of each of the three interventions.

### Relative cost-effectiveness of interventions

The relative cost-effectiveness of the interventions was explored using an indirect comparison of the trials’ efficacy results and calculating the ICER between the two most effective trials.

### Sensitivity analysis

Uni-variate sensitivity analyses were conducted to test plausible variation in the analysis parameters compared to base case ICERs for the interventions with positive ICERs The sensitivity analyses assessed the effect of i) variation in the magnitude of treatment effect using the lower and upper confidence interval limits and ii) variation in costs of intervention strategy 1 (support officers) using the lower and upper bounds of project officer salary.

The three trials were approved by the Hunter New England Area Human Research Ethics Committee (06/07/26/4.04), the University of Newcastle Human Research Ethics Committee (H-2008-0343) and the NSW Department of Education and Communities (DEC) (#2012277).

## Results

### Trial effectiveness

Relative to control groups, schools receiving the ‘high’ and ‘medium intensity’ interventions were significantly more likely to have menus adherent to the policy (RR = 14.41 (95% CI 2.08, 99.97); p = < 0.001 and RR = 4.29(95% CI 1.04, 17.68); *p* = 0.02 respectively). For schools receiving the ‘low intensity’ intervention, the difference in the proportion of schools adherent compared to control schools approached statistical significance (RR = 4.44 (0.65, 30.11); *p* = 0.06) [19]. (Table 2).

**Table 2:** Intention to treat analysis of the three trials primary outcomes (composite): overall compliance

	Baseline		Follow-up		Intervention v Control at follow-up		
	Intervention n (%)	Control n (%)	Intervention n (%)	Control n (%)	Estimated difference % (95%CI)	Relative Risk (95% CI)	*P*-value
High Intensity	0 (0)	0 (0)	21 (60)	2 (5)	56 (35 to 76)	14.41 (2.08 to 99.97)	< 0.001^a^
Medium Intensity	2 (7)	1 (4)	10 (36)	2 (8)	27 (6 to 48)	4.29 (1.04 to17.68)	0.02^a^
Low Intensity	0 (0)	1 (3)	8 (22)	4 (5)	16 (− 1 to 34)	4.44 (0.65 to 30.11)	0.0624

^a^Statistically significant

### Within-trial cost and cost effectiveness

Table 1 shows the total delivery costs for the three interventions, the costs per school, and cost per intervention strategy. The total cost of delivering the ‘high intensity’ intervention was $166,971, the cost for the ‘medium intensity’ intervention was $70,926 and for the ‘low intensity’ intervention $75,682. Adjusting for the duration over which the interventions were conducted, 12 months, 9 months and 12 months, respectively, the cost of the ‘medium’ intensity intervention was scaled to be $94,568. The average cost per school for each of the interventions was $4771 (high intensity), $2216 (medium intensity), and $2102 (low intensity).

**Fig. 1:**
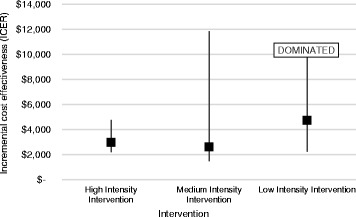
Incremental Cost Effectiveness Ratios for the three trials

Incremental cost effectiveness ratios (ICERs) were calculated as the incremental cost per additional percentage point increase in proportion of schools reporting adherence. The point estimate ICERs for the three interventions versus usual support were $2982 (high intensity), $2627 (medium intensity) and $4730 (low intensity). Figure 1 presents the ICERs and associated uncertainty intervals. The low intensity intervention was excluded from further analysis due to the higher point estimate ICER and dominated upper uncertainty interval, indicative of both higher costs and lower efficacy than usual support. In contrast, the tightness of the uncertainty intervals around the ‘high intensity’ intervention suggests a higher degree of certainty in the effectiveness of that trial.

### Sensitivity analysis

**Fig. 2:**
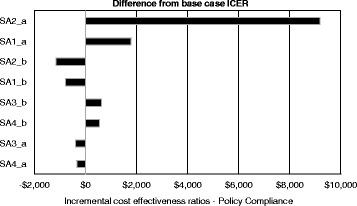
Sensitivity Analysis for high intensity and medium intensity interventions

Figure 2 presents the univariate sensitivity testing results for the ‘high’ and ‘medium intensity’ interventions. The results of the analysis indicate that the ICERs for the ‘high’ and ‘medium intensity’ interventions were most sensitive to the estimate of treatment effect, specifically the lowest bound of the efficacy confidence intervals.

### Relative cost effectiveness of interventions

**Fig. 3:**
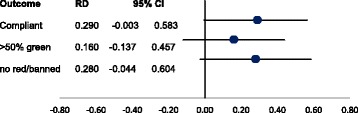
Indirect comparison between High Intensity Intervention and Medium Intensity Intervention

The similarity or homogeneity of the trials in terms of design, setting and outcomes measured supports the validity of using indirect comparison to test the relative cost-effectiveness of the interventions. The indirect comparison between the ‘high’ and ‘medium intensity’ interventions showed no statistically significant difference in efficacy. For the overall compliance outcome, the risk difference between these trials was calculated to be 0.29 (− 0.003, 0.583) (Fig. 3). This result translated into overlapping uncertainty intervals around the ICERs, indicating a strong likelihood that there is no difference in cost-effectiveness between the interventions. However, at a significantly lower overall cost, even when scaled over 12 months, the ‘medium intensity’ intervention would be the optimal choice for policy makers.

## Discussion

This is the first study to assess the cost and cost-effectiveness of three implementation support interventions of varying intensity using similar methods in enhancing the implementation of a healthy school canteen policy, and one of few cost-effectiveness studies of strategies to implement school or community based health promotion initiatives. The ‘high intensity’ intervention incurred the greatest costs per school ($4771/ school), followed by the ‘medium intensity’ intervention ($2216/school) and the ‘low intensity’ intervention ($2102/school). The comparison between the ‘high’ and ‘medium intensity’ interventions showed no statistically significant difference between the two in cost-effectiveness. The results indicate that the ‘medium’ and ‘high intensity’ interventions were potentially cost-effective strategies to support schools to improve implementation of a healthy canteen policy. Such findings provide previously unavailable evidence to inform policy and practice decisions regarding the nature and extent of investment required to achieve the intended public health benefits of school food availability policies.

Cost-effectiveness analyses of implementation strategies in non-clinical settings are not common [26] and to the author’s knowledge, are non-existent with regard to food availability policy interventions in schools. As a consequence, no comparable ICERs were available to place the ICERs of the individual interventions addressed in this study in a broader cost-effectiveness context however, the analyses of three interventions in this study provides a strong basis for future research in this area. Without standardized outcomes for economic evaluation of implementation strategies, comparisons across different interventions are difficult. Similarly, no previous research has reported the relative cost-effectiveness of multiple implementation interventions in improving school adherence with food availability policies or guidelines. Researchers in other disciplines have conducted economic analyses to compare alternative implementation strategies in their field [27, 28] however comparison to ICERs reported in these studies was not plausible due to differences in outcomes.

The on-going support provided by school support staff in the ‘high intensity’ intervention was the largest cost driver (average of $4316 /school). It is likely intensive support contributed to the overall greater effectiveness of the intervention [29]. Text messaging as opposed to intensive on-going support, which included on-site visits, was the major difference in program delivery between the ‘medium’ and ‘high intensity’ interventions and therefore is assumed to have contributed significantly to the lower cost of the ‘medium intensity’ intervention.

The costs and time required for intervention development and set up is likely to be significant. While many of the resources developed for the three trials have the potential to be implemented in other jurisdictions, some adaptation may be required to address local context differences in terms of policy guidelines, availability of appropriate foods and beverages and type of food service provided by schools. Notwithstanding these potential differences the structure and focus of the implementation support strategies are likely to be applicable across jurisdictions.

Limitations of this study include the relative small sample size of each trial and short follow-up period. Secondly, it should be noted that comparisons are indirect only as the interventions were not tested in a single factorial trial. As cost-effectiveness was measured using a health service delivery perspective, opportunity costs to canteen managers, principals or schools were not included in the study. Further, the aggregate nature of the costs does not permit uncertainty analysis considering variation in both costs and outcomes at the school or student level, and the generalizability of the findings to other countries or jurisdictions is unknown.

The translation of the outcomes captured by the three trials into outcomes commonly used for economic evaluations such as DALYs or percent body fat reduction was not possible in this analysis given the study focus on canteen rather than student level outcomes [12, 30]. Interventions targeting school healthy food policy implementation that include individual outcome data capturing child dietary intake may provide policy makers with additional useful information on which to make cost-effectiveness comparisons.

A major strength of the study is that it is based on data collected from rigorous implementation RCTs, minimizing bias, all conducted within the same region, and, using comprehensive menu audits to assess policy adherence. Costs associated with the intervention were collected prospectively thus improving accuracy by eliminating recall bias.

## Conclusion

This study provides the first information regarding the cost-effectiveness of strategies for supporting implementation of school healthy canteen policies and for guiding policy decisions regarding the allocation of scarce resources. Whether such findings are achieved when the strategies are implemented at-scale warrants further research to ensure the benefits of finite health resources return the greatest health benefits to the community.

## References

[1] GBD 2013 Risk Factors Collaborators (2015). Global, regional, and national comparative risk assessment of 79 behavioural, environmental and occupational, and metabolic risks or clusters of risks in 188 countries, 1990–2013: a systematic analysis for the global burden of disease study 2013. Lancet.

[2] Australian Institute of Health and Welfare 2014. Australia’s health 2014. Australia’s health series no. 14. Cat. No. AUS 178. Canberra: AIHW. 2014.

[3] Magarey AMDL, Boulton TJ (2001). Prevalence of overweight and obesity in Australian children and adolescents: reassessment of 1985 and 1995 data against new standard international definitions. Med J Aust.

[4] Serdula MK, Ivery D, Coates RJ, Freedman DS, Williamson DF, Byers T (1993). Do obese children become obese adults? A review of the literature. Prev Med.

[5] Withrow D, Alter DA (2011). The economic burden of obesity worldwide: a systematic review of the direct costs of obesity. Obes Rev.

[6] Katz DL, O'Connell M, Njike VY, Yeh MC, Nawaz H (2008). Strategies for the prevention and control of obesity in the school setting: systematic review and meta-analysis. Int J Obes.

[7] Mayne SL, Auchincloss AH, Michael YL (2015). Impact of policy and built environment changes on obesity-related outcomes: a systematic review of naturally occurring experiments. Obes Rev.

[8] Wolfenden L, Nathan N, Janssen LM (2017). Multi-strategic intervention to enhance implementation of healthy canteen policy: a randomised controlled trial. Implement Sci.

[9] Erdol S, Mazzucco W, Boccia S (2014). Cost effectiveness analysis of childhood obesity primary prevention programmes: a systematic review. Epidemiol Biostat Public Health.

[10] Haby MM, Vos T, Carter R (2006). A new approach to assessing the health benefit from obesity interventions in children and adolescents: the assessing cost-effectiveness in obesity project. Int J Obes.

[11] Wang LY, Gutin B, Barbeau P (2008). Cost-effectiveness of a school-based obesity prevention program. J Sch Health.

[12] Wang LY, Yang Q, Lowry R, Wechsler H (2003). Economic analysis of a school-based obesity prevention program. Obesity.

[13] Hirschman J, Chriqui JF (2013). School food and nutrition policy, monitoring and evaluation in the USA. Public Health Nutr.

[14] Department of Education. Dimbleby H, Vincent J. The School Food Plan. 2013. https://www.gov.uk/government/publications/the-school-food-plan. Accessed 19 Mar 2018.

[15] NSW Department of Health (DoH) Department of Education and Training (DET) (2012). Fresh tastes @ school NSW healthy school canteen strategy: canteen menu planning guide.

[16] U.S. Department of Health and Human Services, Centers for Disease Control and Prevention. Results from the School Health Policies and Practices Study 2012. In: National Center for HIV/AIDS VH, STD, and TB Prevention (Division of Adolescent and School Health). Washington: US Department of Health and Human Services; 2013.

[17] Lawlis T, Knox M, Jamieson M (2016). School canteens: a systematic review of the policy, perceptions and use from an Australian perspective. Nutr Diet.

[18] Nathan N, Yoong SL, Sutherland R (2016). Effectiveness of a multicomponent intervention to enhance implementation of a healthy canteen policy in Australian primary schools: a randomised controlled trial. Int J Behav Nutr Phys Act.

[19] Yoong SL, Nathan N, Wolfenden L (2016). CAFE: a multicomponent audit and feedback intervention to improve implementation of healthy food policy in primary school canteens: a randomised controlled trial. Int J Behav Nutr Phys Act.

[20] NSW Department of Education and NSW Ministry of Health. Live Life Well @ School. 2017. Healthy Kids Website. https://www.healthykids.nsw.gov.au/teachers-childcare/live-life-well-@-school.aspx. Accessed 19 Mar 2018.

[21] Woods J, Bressan A, Langelaan C, Mallon A, Palermo C (2014). Australian school canteens: menu guideline adherence or avoidance?. Health Promot J Aust..

[22] Health Stats NSW. Population growth by Local Health District. 2014. http://www.healthstats.nsw.gov.au/Indicator/dem_pop_lhnmap/dem_pop_lhn_snap. Accessed 19 Mar 2018.

[23] Ardzejewska K, Tadros R, Baxter D (2012). A descriptive study on the barriers and facilitators to implementation of the NSW (Australia) healthy school canteen strategy. Health Educ J.

[24] Pettigrew S, Donovan RJ, Jalleh G, Pescud M (2014). Predictors of positive outcomes of a school food provision policy in Australia. Health Promot Internation.

[25] Reilly K, Nathan N, Wolfenden L (2017). Validity of four measures in assessing school canteen menu compliance with state-based healthy canteen policy. Health Promot J Aust.

[26] Lau R, Stevenson F, Ong BN (2015). Achieving change in primary care--effectiveness of strategies for improving implementation of complex interventions: systematic review of reviews. BMJ Open.

[27] Coudeville L, Van Rie A, Getsios D, Caro JJ, Crepey P, Nguyen VH (2009). Adult vaccination strategies for the control of pertussis in the United States: an economic evaluation including the dynamic population effects. PLoS One.

[28] Kania D, Sangare L, Sakande J (2009). A new strategy to improve the cost-effectiveness of human immunodeficiency virus, hepatitis B virus, hepatitis C virus, and syphilis testing of blood donations in sub-Saharan Africa: a pilot study in Burkina Faso. Transfusion.

[29] Wolfenden L, Nathan N, Williams C (2014). A randomised controlled trial of an intervention to increase the implementation of a healthy canteen policy in Australian primary schools: study protocol. Implement Sci.

[30] Carter R, Moodie M, Markwick A (2009). Assessing cost-effectiveness in obesity (ACE-obesity): an overview of the ACE approach, economic methods and cost results. BMC Public Health.

